# The functional gene composition and metabolic potential of coral-associated microbial communities

**DOI:** 10.1038/srep16191

**Published:** 2015-11-05

**Authors:** Yanying Zhang, Juan Ling, Qingsong Yang, Chongqing Wen, Qingyun Yan, Hongyan Sun, Joy D. Van Nostrand, Zhou Shi, Jizhong Zhou, Junde Dong

**Affiliations:** 1 CAS Key Laboratory of Tropical Marine Bio-resources and Ecology, South China Sea Institute of Oceanology, Chinese Academy of Sciences, Guangzhou 510301, China; 2 Tropical Marine Biological Research station in Hainan, South China Sea Institute of Oceanology, Chinese Academy of Sciences, Sanya 572000, China; 3 Department of Microbiology and Plant Biology, Institute for Environmental Genomics, University of Oklahoma, Norman, Oklahoma 73019, USA; 4 Fisheries College, Guangdong Ocean University, Zhanjiang 524025, China; 5 Institute of Hydrobiology, Chinese Academy of Sciences, Wuhan 430072, China; 6 College of Animal Science, South China Agricultural University, Guangzhou 510642, China

## Abstract

The phylogenetic diversity of coral-associated microbes has been extensively examined, but some contention remains regarding whether coral-associated microbial communities are species-specific or site-specific. It is suggested that corals may associate with microbes in terms of function, although little is known about the differences in coral-associated microbial functional gene composition and metabolic potential among coral species. Here, 16S rRNA Illumina sequencing and functional gene array (GeoChip 5.0) were used to assess coral-associated microbial communities. Our results indicate that both host species and environmental variables significantly correlate with shifts in the microbial community structure and functional potential. Functional genes related to key biogeochemical cycles including carbon, nitrogen, sulfur and phosphorus cycling, metal homeostasis, organic remediation, antibiotic resistance and secondary metabolism were shown to significantly vary between and among the four study corals (*Galaxea astreata*, *Porites lutea*, *Porites andrewsi* and *Pavona decussata*). Genes specific for anammox were also detected for the first time in the coral holobiont and positively correlated with ammonium. This study reveals that variability in the functional potential of coral-associated microbial communities is largely driven by changes in environmental factors and further demonstrates the importance of linking environmental parameters with genomic data in complex environmental systems.

The coral holobiont is a complex system containing the coral polyp and its associated microbial representatives of all three domains: eukaryota, bacteria, and archaea, as well as numerous viruses^1^. The coral host provides niche microhabitats for microorganism: the surface mucus layer, coral tissue and the calcium carbonate skeleton^1^,^2^. Microbes are vital components of the coral holobiont and key players in both healthy and degraded coral reefs^3^,^4^,^5^,^6^. Knowledge of the interactions between corals and their symbiotic microorganisms is useful for preventing the spread of coral disease^1^. Molecular studies over the last decade have uncovered an astonishing diversity of microorganisms associated with different corals^1^. Both coral species-specific^7^,^8^ and site-specific^9^,^10^ microbial communities have been reported. Accumulated evidence supports both site and species specificity of common bacterial associates^11^. One potential explanation is that corals may associate with microbes in terms of function, rather than identical species^12^,^13^. In addition, environmental factors are also considered as drivers of coral-associated bacterial community structure^10^,^14^,^15^,^16^.

Determining the functional role of complex and dynamic coral-associated microbial communities is challenging due to our inability to culture the vast majority (~99%) of microorganisms^1^,^17^. Recently, rapid development of high-throughput metagenomic technologies enabled scientists to discover the potential functional roles of the coral holobiont members. The metagenomic study by Wegley *et al.* (2007)^18^ provides a metabolic snapshot of microbes associated with the reef-building coral *Porites astreoides*. Significant changes were found in functional potential when the coral holobiont was exposed to stress or disease^19^,^20^. However, little is known about the variability of coral-associated microbial functional potential among coral species or the relationships between the functional genes of coral-associated microbial communities and environmental variables. A microarray based metagenomic tool named GeoChip has been widely used for functionally profiling microbial communities from various environments^21^,^22^,^23^,^24^. Its use allows us to comprehensively investigate the functional potentials of microbial communities involved in nitrogen (N), carbon (C), sulfur (S), and phosphorus (P) cycling, metal resistance, antibiotic resistance, organic remediation, and other processes in various ecosystems.

The GeoChip 5.0 (60K arrays) used in this study contained more than 57,000 oligonucleotide probes, covering over 144,000 gene sequences from 393 gene families^25^. Aiming to obtain insights into the associations between microorganisms and corals, four coral species *Galaxea astreata* (GA), *Porites lutea* (PL), *Porites andrewsi* (PA) and *Pavona decussata* (PD) were collected from the Luhuitou fringing reef, South China Sea. High throughput 16S rRNA Illumina sequencing and GeoChip 5.0 were employed to test (1) whether the functional potential of coral-associated microbes play a more important role than taxonomic composition within a coral species and (2) whether microbial functional potential is driven by local environmental factors, given the important role of microbes in driving biogeochemical cycles. Our results indicated that coral-associated microbial communities were metabolically diverse and significantly different among coral species. In contrast to microbial taxonomic composition, environmental factors accounted for the variations of microbial functional potentials.

## Results

### Overall review of coral-associated microbial community

At the taxonomic level, high quality sequences in length of 245–260 bp were kept for subsequent analysis. A total of 319,465 sequences, ranging from 19,100 to 69,870 reads per sample, were obtained from corals GA, PL, PA, and PD (n = 3). Samples were rarefied to 20,000 sequences per sample. All sequences obtained could be assigned to 8,474 operational taxonomic units (OTUs) using the UClust method. These sequences were classified into 23 bacterial phyla. Sequences related to bacteria within the phylum Proteobacteria were the most abundant phyla (35.2–80.7%). Within the Proteobacteria, Gamma- and Alpha- proteobacteria related sequences made up an average of 19.98 ± 4.52% and 21.35 ± 4.79%, respectively (Supplementary Fig. S1).

**Figure 1:**
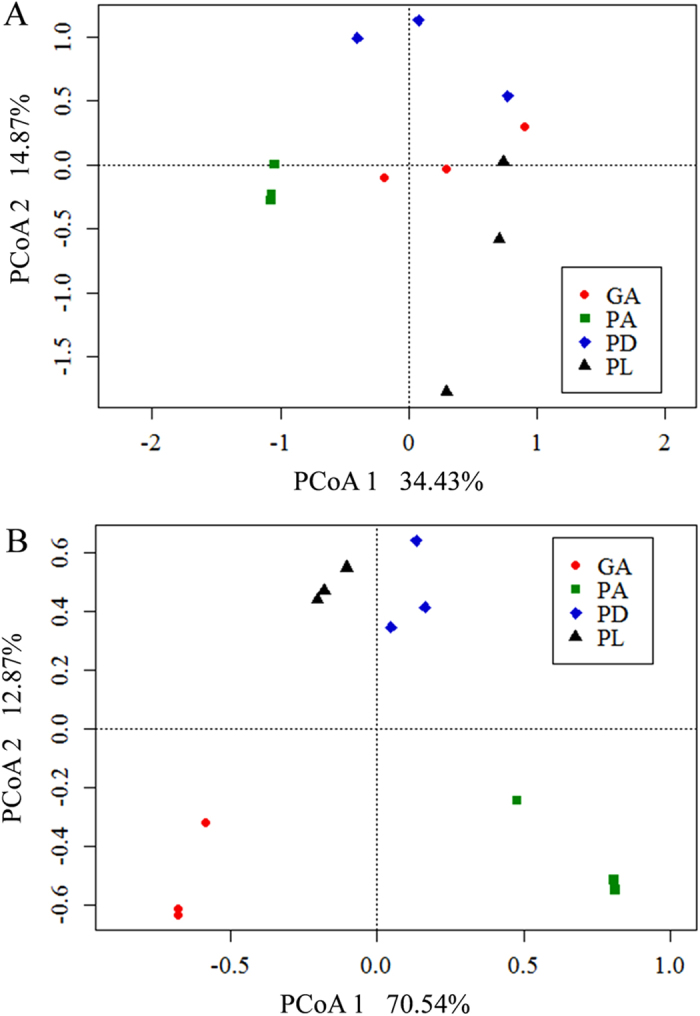
Principal coordinates analysis (PCoA) of microbial community based on high-throughput sequencing data (**A**) and GeoChip data (**B**). The percentage of variation explained by each axis is shown. GA, *Galaxea astreata*. PA, *Porites andrewsi*. PD, *Pavona decussata*. PL, *Porites lutea*.

At the functional gene level, 25,380 gene sequences were detected by GeoChip 5.0 including 22,356 sequences derived from bacteria, 622 from archaea, 1,891 from fungi and the remaining 511 from other unclassified organisms. The overall microbial functional diversity differed significantly based on alpha-diversities (ANOVA, *P* < 0.001) as shown by Shannon-Weaver (H’), Simpson reciprocal indices (1/D) and Simpson evenness (E) (Supplementary Table S1). To further examine differences among coral species, principal coordinates analysis (PCoA) was performed with high-throughput sequencing and GeoChip data, respectively. The microbial community functional potential was significantly different among coral species as revealed by the plot (Fig. 1), which was also verified by the dissimilarity test of the whole GeoChip data (PERMANOVA, *P* < 0.05, Table 1). The number of functional genes detected for individual coral samples ranged from 288 to 348. The top 100 most abundant functional genes were significantly different among coral species (ANOVA, *P* < 0.001, Supplementary Table S2). Generally, profiles derived from coral GA showed the highest gene signal intensities and PA showed the lowest gene signal intensities. Almost one third of the top 100 most abundant functional genes showed a significant difference between coral PD and PL samples (LSD tests, *P* < 0.05, Supplementary Table S2).

### Genes involved in key biogeochemical cycling processes

Identifying microbial functional genes is important to establish linkages between community structure and functions. To understand the functional potential of coral-associated microbial communities, microbial functional gene categories for major biogeochemical/metabolic processes were examined.

**Figure 2:**
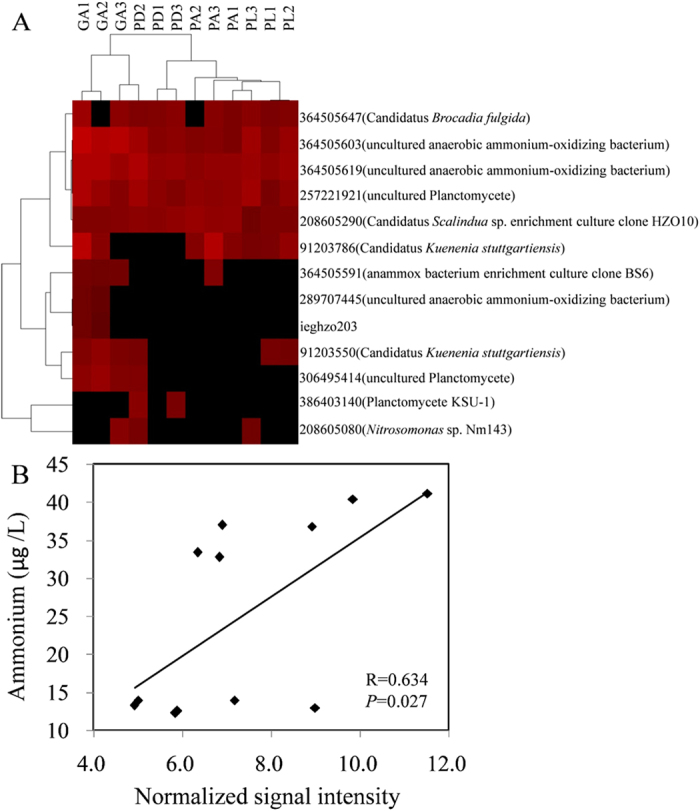
Hierarchical cluster analysis of all genes sequences related to anammox (**A**). Red indicates signal intensities above background, whereas black indicates signal intensities below background. Brighter red coloring indicates higher signal intensities. Correlations between ammonium and the abundance of genes involved in anammox (**B**). The full name of each coral species is given in Fig. 1.

Microbial activity is responsible for the major nitrogen (N) transformations within the coral holobiont. We detected genes involved in denitrification, nitrogen fixation, ammonification, assimilatory N reduction, N assimilation, nitrification and anammox. The majority of genes involved in denitrification and nitrogen fixation originate from unclassified bacteria (Supplementary Fig. S2). Most sequences involved in ammonification were related to bacteria within the phylum Proteobacteria and Actinobacteria. Additionally, archaeal and fungal sequences related to denitrification and ammonification, methanogenic Euryarchaeota genes related to nitrogen fixation were also detected (Supplementary Fig. S2). Most of the microbial community involved in nitrogen cycling process differed significantly between every coral pair (PERMANOVA, *P* < 0.05, Table 1 and Supplementary Table S3). Anammox pathway was suggested to be a predominant source of N_2_ production in marine anoxic environments. The genes *hzsA* and *hzo* involved in anammox were detected in all coral samples. Most of the sequences were from Planctomycetes. Moreover, one *hzo* sequence identified as *Nitrosomonas* sp. Nm143 was detected (Fig. 2A). Pearson's correlation analysis showed that the abundance of genes involved in the anammox pathway was positively correlated with the concentration of ammonium (*P* = 0.027, Fig. 2B).

Microorganisms have critical roles in carbon cycling of the biosphere. Key functional genes for carbon degradation, carbon fixation, methane generation, and methane oxidation were detected in all coral samples. Seven CO_2_ fixation pathways, including the Calvin cycle and alternative pathways were detected (Supplementary Table S4). For the first time bacterial genes involved in 3-hydroxypropionate bicycle, bacterial microcompartments and reductive tricarboxylic acid cycle, and archaeal genes related to 3-hydroxypropionate/4-hydroxybutyrate cycle and dicarboxylate/4-hydroxybutyrate cycle were detected in the coral holobiont (Supplementary Table S4). Based on dissimilarity analysis, the composition of functional potential communities involved in all CO_2_ fixation pathways differed significantly between coral species (PERMANOVA, *P* < 0.05, Supplementary Table S3). The genes involved in degradation of 22 different carbon sources were detected, including starch, chitin, hemicellulose, pectin, cellulose, lignin, cutin, vanillin, phospholipids and terpenes degradation (Supplementary Table S4). Most comparisons of any two coral species showed significant differences in the composition of functional potential communities involved in C degradation (PERMANOVA, *P* < 0.05, Supplementary Table S3). For genes involved in methane oxidation and production, significant differences were detected among coral GA, PA, and PL (PERMANOVA, *P* < 0.05, Supplementary Table S3).

In sulfur cycling, genes related to sulfite reduction, sulfide oxidation, sulfur oxidation, adenylyl sulfate reductase and dimethyl sulfoniopropionate (DMSP) degradation were observed. Both archaeal and bacterial genes were detected related to sulfur cycling. Fungal sulfite reduction gene *sir* was detected in all investigated corals and the sulfite reduction gene fungal *cysJ* was identified in coral GA, The microbial structure and composition related to sulfur cycling differed significantly between every coral pair except for GA and PL (PERMANOVA, *P* < 0.05, Table 1). The sequences detected in phosphorus cycling include genes for phytic acid hydrolysis, polyphosphate degradation and polyphosphate synthesis. Both bacterial and fungal phytase genes were detected. Both archaeal and bacterial *ppk* genes, encoding polyphosphate kinase, which catalyzes polyphosphate synthesis were detected. In addition, bacterial, archaeal and fungal *ppx* genes for polyphosphate degradation were detected. Significant differences were detected in the microbial communities related to phosphorus metabolism between every coral pair (PERMANOVA, *P* < 0.05, Table 1).

We detected 5,526 organic remediation sequences involved in the remediation of aromatics, chlorinated solvents, herbicide related compounds, pesticides related compounds, and other hydrocarbons (Supplementary Table S4). The microbial structure and composition related to organic remediation differed significantly between every coral pair except for PD and PL (PERMANOVA, *P* < 0.05, Table 1 and Supplementary Table S3). Of the 1,853 metal associated genes detected, most were involved in the detoxification of arsenic, tellurium and mercury. Further observation showed that bacterial, archaeal and fungal genes are necessary for antibiotic resistance (detoxification) in arsenic, mercury, and tellurium detoxification were detected (Supplementary Table S4). Significant differences were also detected in the microbial community related to metal homeostasis between every coral pair except for GA and PL (PERMANOVA, *P* < 0.05, Table 1). Archaeal, bacterial and fungal genes were detected related to antibiotics resistance (Supplementary Table S4). The microbial community related to antibiotics resistance differed significantly between every coral pair among coral GA, PA and PD (PERMANOVA, *P* < 0.05, Table 1).

### Relationship between microbial functional gene potential and environmental variables

**Figure 3:**
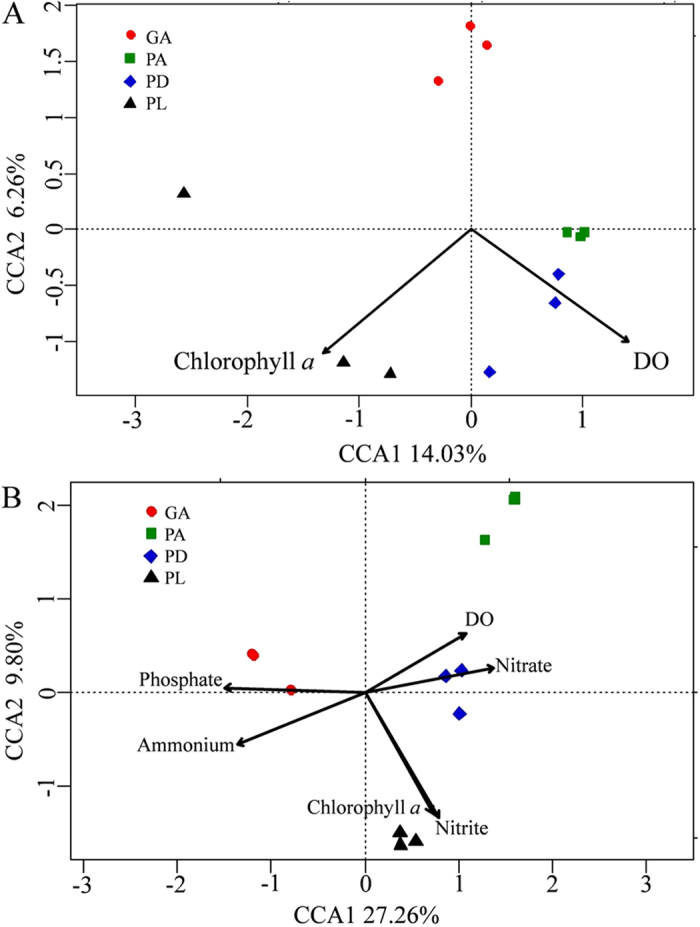
Canonical correspondence analysis (CCA) of high-throughput sequencing data (**A**) and GeoChip data (**B**) with environmental parameters. Environmental variables were chosen based on significance calculated from individual CCA results. The percentage of variation explained by each axis is shown. The full name of each coral species is given in Fig. 1.

Canonical correspondence analysis (CCA) was performed to determine major environmental factors shaping the coral-associated microbial community at the taxonomic or functional gene level. Environmental variables that significantly correlated with the microbial community were selected (Monte Carlo test, P < 0.05) as independent environmental parameters, resulting in significant CCA models by a Monte Carlo permutation test both at the taxonomic (P = 0.01) and functional gene level (P = 0.001). Of all the environmental parameters selected, chlorophyll *a* and dissolved oxygen (DO) were significantly correlated with the microbial community both at the taxonomic level and at the functional level (Monte Carlo test, *P* < 0.05, Supplementary Table S5). The functional gene composition of the microbial community was also significantly correlated with the concentrations of inorganic nitrogen and phosphate (Monte Carlo test, *P* < 0.01, Supplementary Table S5). The correlations visualized by CCA illustrate that the microbial community of PA was positively correlated with the concentration of nitrate and DO both at a taxonomic and a functional level (Fig. 3). However, only at the functional level is the microbial community of coral GA positively correlated with the concentration of ammonium and phosphate, PL positively correlated with the concentration of nitrite and chlorophyll *a* and PD positively correlated with nitrate and DO (Fig. 3B).

**Figure 4:**
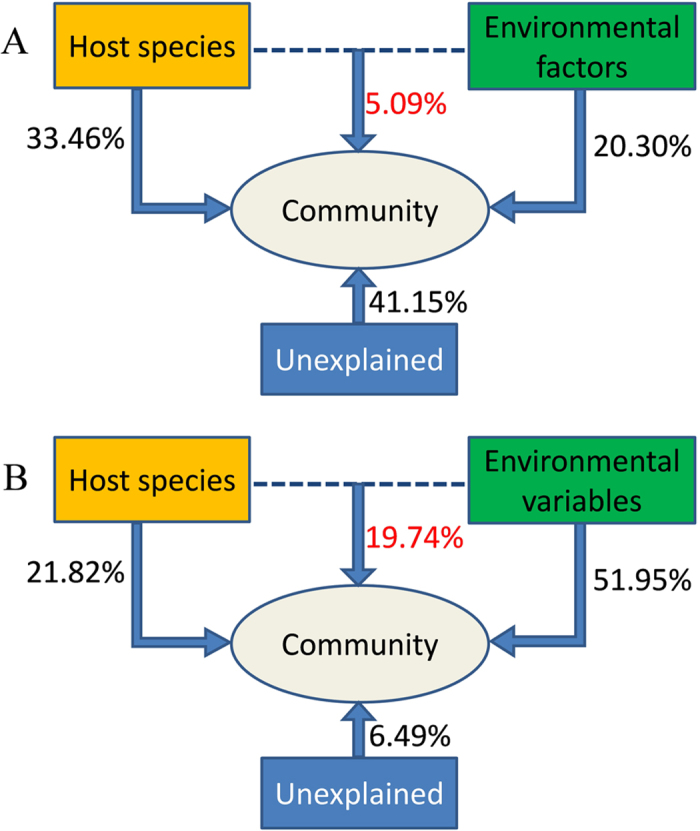
Variance partitioning canonical correspondence analysis (CCA) shows the relative effects of host species and environmental variables on the coral-associated community at taxa level (**A**) and functional gene levels (**B**). The Red marks represent the combined effects from host species and environmental variables. The unexplained represents the effect that could not be explained by neither host species nor environmental variables. Only variables that were significantly correlated with the community (forward selection with Monte Carlo test, P < 0.05) were chosen as environmental variables, Chlorophyll a and DO were chosen as the environmental variables at taxa level, and ammonium, nitrate, nitrite, phosphate, Chlorophyll a and DO were chosen as the environmental variables at functional genes level.

A CCA-based variation partitioning analysis was performed using environmental data and coral species to determine the community variation that can be explained by the environmental variables and host species. Host species and environmental variables showed a significant correlation with the microbial community both at taxonomic and functional gene levels. Host species explained more variations (33.46%) than environmental variables (20.30%) from a taxonomic perspective, whereas environmental variables explained substantially more variation (51.95%) than host species (21.82%) from a functional gene perspective (Fig. 4). Approximately 6.49% of the community functional variation based on Geochip data remained unexplained by the selected variables, which is much lower than the unexplained variability in the taxonomic community (41.15%). While the interaction of both host species and environmental variables explained 5.09% and 19.74% at the taxonomic and functional gene levels respectively (Fig. 4).

## Discussion

There is increasing evidence that coral microbiota are crucial to ocean biogeochemical cycles and control the health and resilience of coral reef ecosystems^26^,^27^. Coral reefs often reside in oligotrophic waters and it has been suggested that the coral-associated microbes supply nutrients for the coral animal. Results from this study showed that 288 to 348 functional genes were detected from individual coral samples related to key biogeochemical cycling processes, including carbon, nitrogen, sulfur and phosphorus cycling, metal homeostasis, organic remediation, antibiotic resistance and secondary metabolism. Functional genes involved in carbon cycling were most abundant in all samples followed by genes involved in organic remediation. Many of the functional genes were of varied origin (archaea, bacteria, fungi and other eukaryota), further supporting that the coral-associated microbe is abundant and diverse. Compared with previous metagenomic studies of the coral holobionts^18^,^19^, our study detected more functional potential in the coral holobiont than previously recognized (Supplementary Table S4), which might be attributed to the differences in technique or coral species. Coral GA showed the highest gene numbers and signal intensities, whilst the lowest were detected in PA samples, consistent with 16S rRNA sequencing results that detected the highest OTU numbers in GA and the lowest in PA coral samples. The microbial functional potential showed significant differences between every coral pair except for PD and PL (PERMANOVA, *P* < 0.05, Table 1). However, significant differences were detected between PD and PL in 18 of all 30 functional potential pathways (PERMANOVA, P < 0.05, Table S3). By contrast, most taxonomic communities did not differ significantly between every coral pair (PERMANOVA, P > 0.05, Table 1). Our results are consistent with previous reports^25^ that the functional potential showed a higher functional diversity than that calculated from OTU composition, suggesting that particular species show distinct functional potential in response to environmental variation.

**Figure 5:**
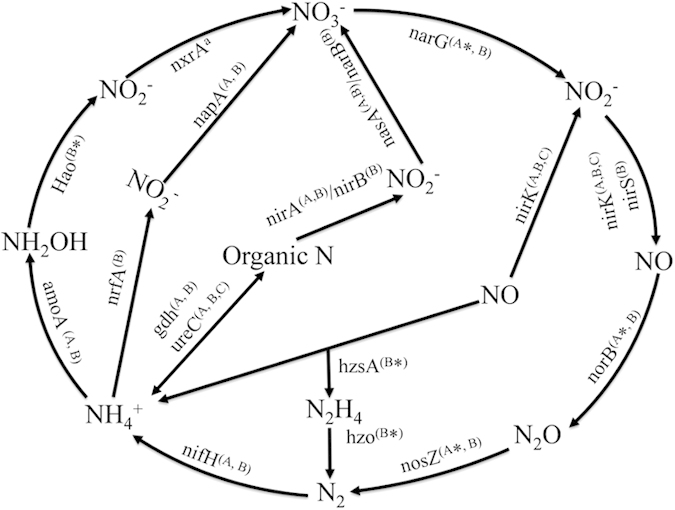
Nitrogen cycling in the coral-associated microbial community. The superscript letters ‘A’ (archaea), ‘B’ (bacteria) and ‘C’ (fungi) indicate the microbial communities identified for each category. *The genes were first detected in the coral holobiont. ^a^The gene cannot be detected.

By trying to unravel the uptake, exchange and transformation of nitrogen in the coral holobiont, insight may be gained into how corals adapt to dynamic environmental conditions of near-shore systems^28^,^29^. In this study, several genes related to the nitrogen cycle were detected for the first time in the coral holobiont (Fig. 5). It is worth noting that genes specific for anammox were detected. This process is catalyzed by a specialized group of planctomycetes^30^. Direct tracer evidence has shown that the anammox pathway, rather than conventional denitrification, can be a significant and potentially predominant source of N_2_ production in marine sediments and anoxic water column systems^31^,^32^,^33^. Globally, this process may be responsible for 30–50% of N_2_ production in the oceans^34^,^35^. However, the importance of anammox in coral reef environments has not yet been considered^29^. Higher inorganic nitrogen concentrations provoke negative responses on the coral holobionts, which ultimately promote coral reef decline^36^. How corals adapt to higher inorganic nitrogen concentrations of near-shore systems remains unknown. In this study, genes related to anammox were detected, indicating that ammonium and nitrite can be converted directly into dinitrogen gas via anammox pathways in the coral holobint. Moreover, positive correlation was detected between the concentration of ammonium and the abundance of anammox genes, suggesting higher abundance of anammox genes may reflect the higher concentration of ammonium at these high-nutrient sites. However, further tracer study on N_2_ production of anammox pathway in the coral holobiont will help us understand the coral ecosystem nitrogen cycle.

Traditionally, it was thought that carbon cycling in the coral holobiont was characterized with respect to the coral-zooxanthellae relationship^37^. However, current evidence reveals that the abundant and diverse prokaryotic communities associated with the coral holobiont also play an important role in carbon cycling through multiple processes^18^,^19^. Our study highlights the potential for carbon cycling in the coral holobiont through the identification of respective microbial genes. A lot of bacterial and archaeal genes involved in carbon cycling were detect for the first time in the coral holobiont (Supplementary Table S3) suggesting that bacteria and archaea play significant roles in the carbon cycle may be severely underestimated^18^,^19^,^38^,^39^,^40^. Metabolism of both organic sulfur and inorganic sulfur is important to the health of the coral holobiont. Recent reports reveal that the metabolism of dimethylsulphoniopropionate (DMSP) in the coral holobionts plays an important role in response to coral thermal stress^41^. Here, bacterial and archaeal genes related to DMSP degradation were detected, suggesting a potential response of microbe to thermal stress. Our results support the previous results that both archaea and bacteria may play a role in sulfur cycling within the coral holobiont (Table S4)^19^. Additionally, the fungal sulfite reduction genes *sir* and *cysJ* were detected, suggesting the coral-associated fungi could be responsible for coral sulfite reduction. Phosphorus cycling in the coral holobiont has not yet been characterized. Genes related to recycling of organic phosphorus, polyphosphate synthesis and degradation were detected in the coral holobiont. Our study enhances the understanding of phosphorus cycling in the coral holobiont. Microorganisms have developed various strategies for metal homeostasis and resistance, including transport systems, resistance proteins and efflux mechanisms^19^. Here, the genes related to the oxidation, reduction and sequestration of arsenic, chromium, copper, mercury and tellurium were detected. Both bacterial and archaeal genes related to mercury detoxification and bacterial genes involved in tellurium detoxification have been documented^19^. However, in our study, bacterial, archaeal and fungal genes necessary for mercury and tellurium detoxification were detected, indicating that all three microbial communities could play a role in metal homeostasis in the coral holobiont. Coastal pollution is one major threat to reefs. There is evidence that coral microbial communities harbor a diverse range of genes related to organic degradation processes that benefit from the resulting elimination of potentially harmful compounds, as well as the addition of potential carbon sources for metabolism^19^. Here, we observed that archaeal and fungal genes are related to the degradation of chlorinated solvents and that fungal genes are necessary for herbicide and pesticide related compound degradation, indicating all three microbial communities play a much greater role in the organic remediation pathway in the coral holobiont than previously known.

Understanding the factors that influence microbial community structure is key study area in microbial ecology. As environmental factors are predicted to select specific metabolic pathways in microbes, environmental factors are usually considerable drivers of the composition and metabolism of reef associated microbial communities^9^,^13^,^15^,^16^. Here, we tease out the key environmental factors driving the structure of coral-associated microbial communities at the taxonomic and functional gene levels. There is evidence that coral associated bacterial community structure was influenced by seawater DO, and several bacterial groups such as *Silicibacter*, *Stenotrophomonas* and *Vibrio* were positively correlated with elevated DO^42^. The significant influence of DO and chlorophyll *a* on the microbial communities was observed both at the taxonomic and functional levels in this study. Nutrient levels have previously been postulated to influence microbial community structure^16^. Microbial communities on reefs with higher nutrient availability were enriched in genes involved in nutrient-related metabolisms^16^. Here, we showed that the concentrations of inorganic nitrogen and phosphate have significant linkages with microbial functional potential. However, the influence was not significant on the taxonomic community structure. This seems to imply that functional gene patterns may be more sensitive to environmental nutrient levels than taxonomic composition. The discordance of taxonomic and functional communities might be the adaptation of microbe to local conditions. Previous reports suggested that adaptation of microbe to local conditions is facilitated by the horizontal transfer of genes responsible for specific metabolic capabilities^16^. In contrast to taxonomic community structure, the ecologically relevant metabolic capabilities of these communities reflected local nutrient concentrations. In addition, host species showed a significant correlation with the microbial community structure both at taxonomic and functional potential levels. In contrast to environmental variables, host species explained relatively more variations at the taxonomic level than at the functional gene level (Fig. 4). To a certain degree our results are consistent with previous metagenomic studies^16^ demonstrating that benthic macroorganisms strongly influence the taxonomic composition of the microbial community, whereas metabolic specialization genes carried by these taxa reflect functional adaptations to oceanographic conditions. It is notable that more variability (48.06%) was unexplained by the host species or environmental variables investigated in this study at a taxonomic level. Other unknown factors such as seasonal changes, geography, rainfall and sunlight intensity suggest further investigation be involved in future studies^9^,^13^,^14^,^15^.

Many functional processes in the coral holobiont are oxygen sensitive such as nitrogen fixation and anammox. Oxygenic photosynthesis renders most of the coral interior oxic during the day, however diurnal patterns of anoxia/hypoxia in the polyp gastrovascular cavity^43^, coral surface (mucus layer)^44^,^45^, skeleton^46^, and in coral tissues compromised by contact with stagnant water or sediment^47^, enables anaerobic forms of bacterial respiration and fermentation to occur within the holobiont. In this study, GA have particular involvement in ammonium and phosphate cycling based on CCA and heatmap results. This may be attributed to Galaxea corals having a deep corallite and gut cavity with significantly less water flow creating an ideal microniche for types of functions that require anoxic conditions. However little is known about the relationship between coral morphology and coral associated microbial community structure.

In conclusion, our results reveal that both host species and environmental variables show significant correlation with the microbial taxonomic community and functional potential. In contrast to microbial taxonomic composition, environmental variables account for the majority of the variations in microbial functional potential. Many functional genes were uncovered in the coral holobiont for the first time, suggesting the coral holobiont can adapt to varying environments via a more comprehensive functional system. However, it should be noted that the presence of a functional gene or gene fragment does not necessarily imply functionality. Therefore, *in situ* or expression-based studies such as mRNA-based microarray hybridization and metagenomic studies are definitely required to elucidate the role that microbes play in the coral holobiont. In addition, further study on relationship between coral morphology and microbial functional potential will provide a better understanding of the difference of functional microorganism among coral species.

## Methods

### Coral sample collection

All coral samples were collected from the Luhuitou fringing reef (18°12'19"N, 109°28'27"E), located in Sanya, South China Sea using a punch and hammer. *Galaxea astreata* (GA) and *Porites lutea* (PL) samples were collected in April in 2013, and *Porites andrewsi* (PA) and *Pavona decussata* (PD) were collected in June in 2013. Three healthy coral colonies from each coral species were collected at a depth of 5–10 m. Triplicate coral fragments (~2 cm2) for each coral colony were collected from the center of each colony and placed in sealed plastic, rinsed thoroughly with sterile seawater at the surface, placed on ice and transported to the laboratory (Tropical Marine Biological Research station in Hainan). Samples were cryopreserved at −20 °C.

### Environmental parameter analysis

Seawater samples within 20 cm of the coral colonies (n = 3) for all environmental parameter analyses were collected using 5 liter GOFLO bottles. Environmental parameters were analyzed as described previously^48^. Dissolved oxygen (DO) was determined by DO meter, pH was measured using a standard hydrogen electrode and reference electrode, and chemical oxygen demand (COD) was determined by alkaline potassium permanganate method. Inorganic nutrient including nitrate, ammonium, nitrite, and phosphate were measured using standard methods as described previously^48^. Triplicates of 1.5 liter water samples from every coral species were filtered through 0.45 μm GF/F filters and deep frozen immediately at −20 °C for chlorophyll a analysis. The chlorophyll a was extracted in 10 ml 90% acetone in the dark for 24 h in a refrigerator and its concentration determined with fluorescence method^48^.

### DNA extraction, purification and quantitation

The coral fragments were suspended in TE buffer, and homogenized in a sterilized mortar and pestle with liquid nitrogen. All of the homogenized solution was transferred to a clean tube, and the total community DNA was extracted using an E.Z.N.A.^®^ Soil DNA Kit (Omega Biotek) according to the manufacturer’s instructions. The DNA was then purified with a Promega Wizard DNA clean-up system (Madison, WI, USA) according to the manufacturer’s directions. DNA quality was evaluated by determining the absorbance ratios of A260/A280 and A260/A230 using a NanoDrop ND-1000 spectrophotometer (NanoDrop Technologies Inc., Wilmington, DE, USA). The final DNA concentrations were quantified by PicoGreen, using a FLUO star Optima instrument (BMG Labtech, Jena, Germany). Purified DNA was stored at −80 °C until used.

### Illumina sequencing and data processing

The V4 region of the 16S rRNA genes was amplified with the primer pair 515F (5′-GTGCCAGCMGCCGCGGTAA-3′) and 806R (5′-GGACTACHVGGGTWTCTAAT-3′) combined with Illumina adapter sequences, and barcode sequences^49^. Sample libraries were generated from purified PCR products. The Miseq 300 cycles kit was used for 2 × 150 bp paired-ends sequencing on Miseq machine (Illumina, San Diego, CA, USA). Refer to supporting information for detailed procedures of PCR amplification, purification and library preparation. Raw sequences were separated to samples using barcodes and with permission of up to one mismatch. Quality trimming was done using Btrim^50^. Forward and reverse reads were merged into full length sequences by FLASH^51^. Sequences were removed if they were too short or contained ambiguous bases. Random re-sampling was performed with 20,000 sequences per sample. The operational taxonomic units (OTUs) were classified using UCLUST at the 97% similarity level. Samples were rarefied to 20,000 sequences per sample. The OTUs that were only present in a single sample were removed. The taxonomic assignment was conducted by RDP classifier (release 5.0)^52^ with minimal 50% confidence estimates.

### GeoChip analysis

The new generation of functional gene array, GeoChip 5.0, was used to analyze the functional potential of coral-associated microbial communities. The purified DNA (500 ng) was labelled with Cy 3 as described previously^24^. The labeled DNA was then re-suspended in hybridization solution [42 μl; 1 × HI-RPM hybridization buffer, 1 × Acgh blocking, 0.05 μg/μl Cot-1 DNA, 10 pM universal standard DNA and 10% formamide (final concentrations)]. GeoChip hybridization was carried out at 67 °C in an Agilent hybridization oven for 24 h. After hybridization, the slides were washed with Agilent Wash Buffers I and II for 5 min and 1 min, respectively. The arrays were then scanned with a NimbleGen MS200 Microarray Scanner (Roche NimbleGen, Inc., Madison, WI, USA). The images were extracted by the Agilent Feature Extraction program.

The raw microarray data was submitted to the GeoChip Microarray Data Manager pipeline (http://ieg.ou.edu/microarray/), and processed as previously described^24^. Poor quality spots or those with a signal-to-noise ratio of less than 2.0 were removed; the positive signals were normalized within each sample and across all samples; and then any spots only detected in one sample were removed. The processed data was then used for further analysis.

### Statistical analysis

The high-throughput sequencing data and GeoChip data were further analyzed with the following statistical methods: (i) The diversity was calculated using Simpson’s 1/D, Shannon-Weiner’s H’, and evenness; (ii) Statistical differences among the microbial diversities from the different coral species were analyzed by a one-way analysis of variance (ANOVA) and least-significant-difference (LSD) tests. A significance level of P < 0.05 was adopted for all comparisons; (iii) Principal coordinates analysis (PCoA) was used to visualize the changes of overall microbial community sturcture; (iv) Dissimilarity test by permutational multivariate analysis of variance (PERMANOVA) were performed with Bray-Curtis using logarithm transformed data for comparing each dataset or sub-dataset of Geochip data. (v) Canonical correspondence analysis (CCA) was used to link the variation within the taxonomic and functional potential community to the explanatory environmental variables; (vi) Partial CCA was used to determine the community variation that can be explained by environmental variables and coral host species (nominal variables). (vii) Monte Carlo permutation was used to test the significance of statistics. All analyses were performed using the Vegan package (v.2.2–0) in R software version 3.1.2 (The R Foundation for Statistical Computing).

## Additional Information

**How to cite this article**: Zhang, Y. *et al.* The functional gene composition and metabolic potential of coral-associated microbial communities. *Sci. Rep.* **5**, 16191; doi: 10.1038/srep16191 (2015).

## Supplementary Material

Supporting Information

## Figures and Tables

**Table 1:** Significance tests of the differences of the microbial communities between any two coral species using Bray–Curtis distances matrices.

	GA vs PA	GA vs PD	GA vs PL	PA vs PD	PA vs PL	PD vs PL
	F (*P*)^1^	F (*P*)	F (*P*)	F (*P*)	F (*P*)	F (*P*)
Taxonomic level						
Whole communities	**4.44 (0.001)**	1.37 (0.206)	1.05 (0.288)	**4.81 (0.001)**	**4.45 (0.001)**	1.72 (0.136)
Functional level						
Whole communities	**37.41 (0.001)**^2^	**27.67 (0.022)**	**17.69 (0.001)**	8.84 (0.051)	**15.03 (0.019)**	**5.98 (0.017)**
Nitrogen cycling	**32.98 (0.006)**	33.72 (0.055)	**17.5 (0.013)**	**8.17 (0.038)**	**14.04 (0.031)**	6.90 (0.084)
Carbon cycling	**36.83 (0.033)**	**25.66 (0.001)**	**17.76 (0.032)**	**8.7 (0.001)**	**14.71 (0.001)**	5.74 (0.087)
Sulfur cycling	**38.14 (0.001)**	**27.61 (0.006)**	**16.57 (0.016)**	**9.21 (0.001)**	**14.39 (0.001)**	5.72 (0.083)
Phosphorus cycling	**35.93 (0.007)**	**25.58 (0.001)**	**17.92 (0.001)**	**8.77 (0.001)**	**15.33 (0.015)**	**5.90 (0.03)**
Organic remediation	**41.97 (0.045)**	**29.79 (0.016)**	**17.83 (0.009)**	**9.57 (0.001)**	**16.79 (0.033)**	6.24 (0.071)
Metal resistance	**37.67 (0.033)**	**25.2 (0.017)**	16.91 (0.052)	**8.86 (0.042)**	**14.97 (0.033)**	**5.64 (0.013)**
Antibiotic resistance	**31.01 (0.023)**	**29.25 (0.019)**	**21.34 (0.009)**	**7.59 (0.001)**	12.56 (0.053)	6.07 (0.083)
Secondary metabolism	**16.7 (0.007)**	**31.17 (0.035)**	**43.33 (0.014)**	**5.13 (0.005)**	**8.28 (0.001)**	2.26 (0.06)

^1^Significance tests were performed by F test based on sequential sums of squares from permutations of the GeoChip hybridization data. P values are of corresponding significance tests.

^2^Significant differences (P < 0.05) are indicated in italic. GA, *Galaxea astreata*. PA, *Porites andrewsi*. PD, *Pavona decussata*. PL, *Porites lutea*.
