# Supplementary material for: The functional gene composition and metabolic potential of coral-associated microbial communities
Source: Sci Rep. 2015 Nov 5;5:16191. doi: 10.1038/srep16191 (PMC4633650; doi:10.1038/srep16191)
Supplement: Supporting Information [file srep16191-s1.pdf]

Supplementary Information

The functional gene composition and metabolic potential of coral-associated microbial communities

Yanying Zhang[1, 2, 3]; Juan Ling[1]; Qingsong Yang[1]; Chongqing Wen[3, 4]; Qingyun Yan[3, 5]; Hongyan Sun[1, 2]; Joy D. Van Nostrand[3]; Zhou Shi[3]; Jizhong Zhou[3]; Junde Dong[1, 2, *]

[1]CAS Key Laboratory of Tropical Marine Bio-resources and Ecology, South China Sea Institute of Oceanology, Chinese Academy of Sciences, Guangzhou 510301, China;

[2]Tropical Marine Biological Research station in Hainan, South China Sea Institute of Oceanology, Chinese Academy of Sciences, Sanya 572000, China;

[3]Department of Microbiology and Plant Biology, Institute for Environmental Genomics, University of Oklahoma, Norman, Oklahoma 73019, USA;

[4]Fisheries College, Guangdong Ocean University, Zhanjiang 524025, China;

[5]Institute of Hydrobiology, Chinese Academy of Sciences, Wuhan 430072, China

[*]Corresponding Author: dongjd@scsio.ac.cn

PCR amplification and sequencing

PCR amplification was performed in 25 μlreactions containing 2.5μl 10×AccuPrime PCR buffer Ⅱ(including dNTPs) (Invitrogen, Grand Island, NY), 0.4 μM of both forward and reverse primers, 10 ng template DNA and 0.2UAccuPrime High FidelityTaq Polymerase. Triplicates of amplification were made for each sample and mixed after PCR amplification to minimize potential biases from amplification [1]. Thermal cycling conditions were as follows: initial denaturation at 94°C for 1 min, followed by 30 cycles of 94°C for 20 s, 53°C for 25 s, and 68°C for 45 s, with final extension at 68°C for 10 min. A total of 200 ng PCR product from each sample was pooled together and purified through QIAquick Gel Extraction Kit (Qiagen, Valencia, CA). Sample libraries for sequencing were prepared according to the MiSeqTM Reagent Kit Preparation Guide (Illumina, San Diego, CA, USA) and the protocol described previously [2,3]. Briefly, sample denaturation was performed by mixing 10 μl of combined PCR products (2 nM) and 10 μl 0.1 M NaOH. Denatured DNA was diluted to 8 pM and mixed with equal volume of 8 pMPhix library. A total of 600 μl sample mixture, together with customized sequencing primers for forward, reverse, and index reads, were loaded into the corresponding wells on the reagent cartridge of a Miseq 300 cycles kit and run on Miseq for 2x150 bp paired-ends sequencing (Illumina, San Diego, CA).

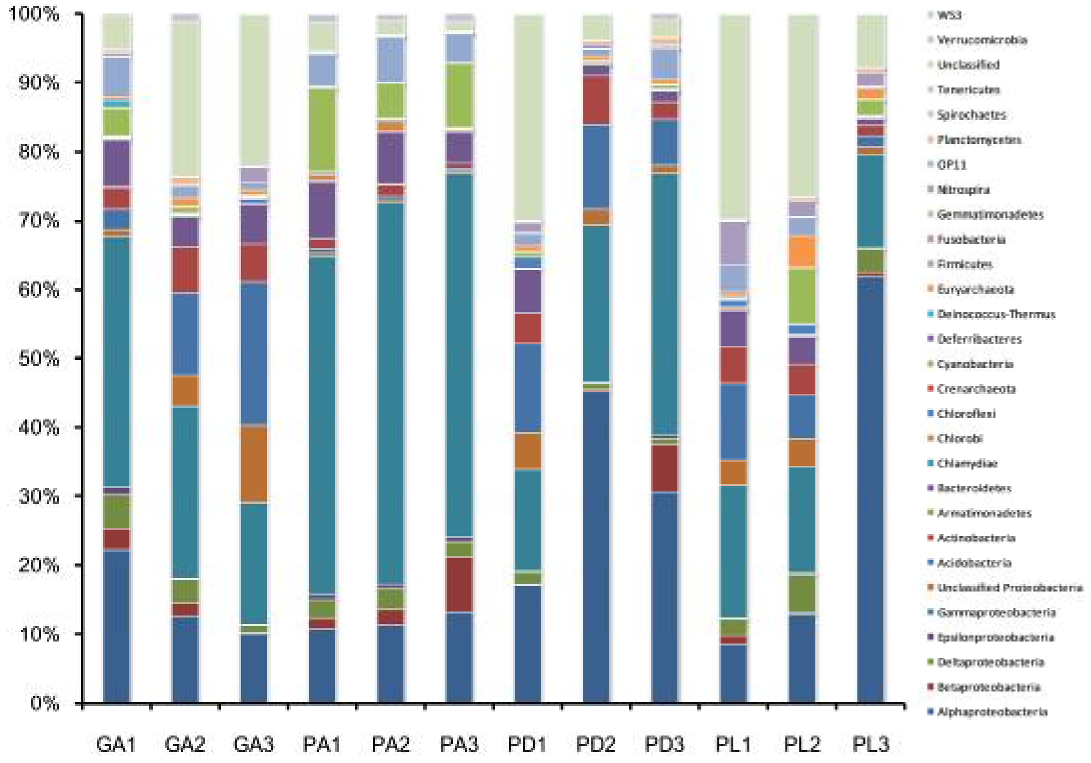

Figure S1. Microbial composition profiles of all coral samples. Microbial taxa were categorized at the phylum level except for the Proteobacteria, which were categorized by class. GA, *Galaxea astreata*. PA, *Porites andrewsi*. PD, *Pavona decussata*. PL, *Porites lutea*.

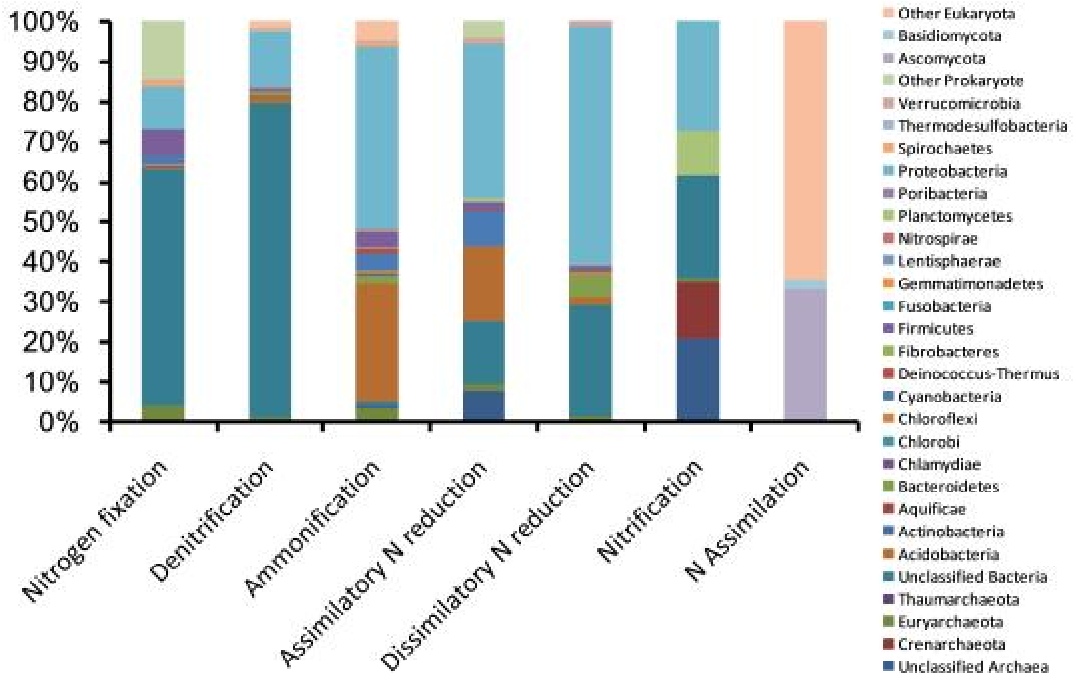

Figure S2 Microbial composition profiles of nitrogen cycling. Microbial taxa were categorized at the phylum level. Eukaryota except fungi were designated as "Other Eukaryota ".

Table S1 The number of detected gene sequences and diversity indices of coral samples (given are the mean value and standard error, SE).

	***Galaxea astreata* Mean±SE.**	***Porites andrewsi* Mean±SE**	***Pavona decussata* Mean±SE**	***Porites lutea* Mean±SE**	**Whole communities *P*-value[1]**
**Taxonomic level**					
OTUs	1891±252a[2]	910±68b	1243±64bc	1522±254ac	0.030
Shannon (H')	5.60±0.26a	4.41±0.09b	4.65±0.12ab	4.84±0.64ab	0.176
Simpson (1/D)	64.19±12.89a	21.84±1.17b	30.59±6.27ab	45.28±19.46ab	0.149
Simpson E	0.03±0.00	0.02±0.00	0.02±0.01	0.03±0.01	0.808
**Functional level**					
No. genes[3]	343±5a	294±4b	318±1c	319±2c	<0.001
Shannon (H')	10.03±0.06a	8.73±0.13b	9.26±0.04c	9.43±0.03d	<0.001
Simpson (1/D)	22362±1204a	6283±862b	10475±427c	12330±382d	<0.001
Simpson E	0.97±0.002a	0.99±0.001b	0.98±0.001c	0.98±0.0004d	<0.001

[1]P values from the ANOVA among all coral species. [2]Different letters indicate statistical differences at a *P* value of <0.05 among coral species by least-significant-difference (LSD) tests. [3]The numbers of functional genes detected.

Table S2 Normalized signal intensities (given are the mean value and standard error, SE) of the top 100 most abundant functional genes determined by GeoChip 5.0.

**Gene**	**Gene category**	**Subcategory**	***Galaxea astreata*** **Mean±SE.**	***Porites andrewsi*** **Mean±SE**	***Pavona decussata*** **Mean±SE**	***Porites lutea*** **Mean±SE**	**Whole communities P-value[1]**
amyA	Carbon Cycling	Carbon degradation	2265.85±121.17a[2]	704.34±83.41b	1109.25±61.21c	1274.69±24.8c	<0.001
chitinase	Carbon Cycling	Carbon degradation	518.92±32.57a	149.71±21.22b	246.85±11.62c	282.44±13.4c	<0.001
acetylglucosaminidase	Carbon Cycling	Carbon degradation	352.15±18.48a	108.18±14.79b	179.28±8.43c	191.38±8.43c	<0.001
ara	Carbon Cycling	Carbon degradation	336.81±19.91a	90.24±14.73b	168.11±8.7c	184.48±3.69c	<0.001
xylanase	Carbon Cycling	Carbon degradation	289.41±14.48a	87.92±16.45b	137.49±7.92c	164.42±7.28c	<0.001
cellobiase	Carbon Cycling	Carbon degradation	259.96±18.51a	60.72±9.47b	109.2±5.83c	129.45±1.81c	<0.001
phenol oxidase	Carbon Cycling	Carbon degradation	252.68±16.73a	63.52±7.21b	109.06±5.31c	126.39±5.01c	<0.001
cutinase	Carbon Cycling	Carbon degradation	208.37±7.5a	65.36±9.5b	106.65±3.19c	136.97±1.63d	<0.001
xyla	Carbon Cycling	Carbon degradation	190.03±8.83a	44.07±5.8b	85.73±4.58c	110.6±2.94d	<0.001
endochitinase	Carbon Cycling	Carbon degradation	177.79±10.78a	46.86±7.07b	78.23±2.76c	89.02±2.39c	<0.001
mannanase	Carbon Cycling	Carbon degradation	152.69±5.48a	37.72±4.96b	71.15±4.6c	83.22±5.8c	<0.001
endoglucanase	Carbon Cycling	Carbon degradation	144.69±8.28a	44.08±6.11b	70.74±4.08c	80.76±1.23c	<0.001
RgaE	Carbon Cycling	Carbon degradation	139.5±7.28a	44.99±4.53b	74.23±4.35c	80.74±4.36c	<0.001
vana	Carbon Cycling	Carbon degradation	132.32±4.74a	45.13±6.52b	72.29±3.62c	82.15±0.99c	<0.001
rgl	Carbon Cycling	Carbon degradation	121.91±4.78a	39.36±4.51b	64.67±2.21c	71.08±3.57c	<0.001
pme	Carbon Cycling	Carbon degradation	122.62±8.04a	34.47±6.12b	59.05±2.49c	67.14±3.31c	<0.001
pectinase (pectate lyase)	Carbon Cycling	Carbon degradation	114.08±8.33a	33.67±4.98b	54.48±3.54c	62.72±3.46c	<0.001
glucoamylase	Carbon Cycling	Carbon degradation	113.73±5.57a	33.04±7.31b	50.01±3.39c	57.51±3.07c	<0.001
cda	Carbon Cycling	Carbon degradation	108.43±5.37a	33.39±4.61b	56.29±3.93c	54.55±2.76c	<0.001
alginase	Carbon Cycling	Carbon degradation	96.35±5.16a	30.85±4.57b	48.14±1.67c	61.04±2.66d	<0.001

Table S2 (Continue)

Gene	Gene category	Subcategory	*Galaxea astreata* Mean±SE.	*Porites andrewsi* Mean±SE	*Pavona decussata* Mean±SE	*Porites lutea* Mean±SE	Whole communities P-value[1]
exoglucanase	Carbon Cycling	Carbon degradation	91.21±5.18a	20.89±4.06b	35.45±2.3c	39.8±2.39c	<0.001
pula	Carbon Cycling	Carbon degradation	79.82±6.17a	25.18±3.28b	37.77±3.81bc	40.77±1.41c	<0.001
limeh	Carbon Cycling	Carbon degradation	66.39±2.98a	15.98±3.44b	29.63±1.61c	36.53±0.34c	<0.001
pec Cdeg	Carbon Cycling	Carbon degradation	61.24±3.73a	16.21±1.94b	29.26±2.38c	36.19±1.26c	<0.001
tktA	Carbon Cycling	Carbon fixation	364.29±21.78a	96.74±15.55b	172.55±5.4c	206.63±2.66c	<0.001
fthfs	Carbon Cycling	Carbon fixation	322.36±23.83a	78.57±12.07b	141.84±8.75c	165.89±8.71c	<0.001
FBPase	Carbon Cycling	Carbon fixation	214.23±10.01a	57.33±6.25b	103.09±5.28c	119.27±4.93c	<0.001
CsoS1 CcmK	Carbon Cycling	Carbon fixation	214.72±14.86a	65.59±8.29b	101.14±4.24c	104.58±5.08c	<0.001
rubisco	Carbon Cycling	Carbon fixation	207.45±13.07a	53.55±6.3b	92.98±2.8c	109±5.14c	<0.001
GAPDH Calvin	Carbon Cycling	Carbon fixation	208.93±12.61a	50.45±10.91b	89.81±3.61c	108.69±2.69c	<0.001
TIM	Carbon Cycling	Carbon fixation	179.04±8.58a	61.37±9.52b	96.74±5c	114.73±5.16c	<0.001
PRI	Carbon Cycling	Carbon fixation	145.53±10.21a	36.82±5.09b	75.63±3.1c	87.96±3.38c	<0.001
ccmL	Carbon Cycling	Carbon fixation	128.56±8.24a	38.78±6.04b	59.83±3.88c	70.4±1.72c	<0.001
pgk	Carbon Cycling	Carbon fixation	131.78±6.23a	30.83±2.97b	52.66±1.76c	69.61±2.33d	<0.001
FBP aldolase	Carbon Cycling	Carbon fixation	101.37±5.03a	22.16±2.68b	36.97±1.62c	46.3±1.56c	<0.001
PRK	Carbon Cycling	Carbon fixation	68.53±4.68a	19.06±3.88b	25.18±1.7c	35.28±1.07c	<0.001
mcra	Carbon Cycling	Methane	94.44±7.23a	20.32±3.99b	34.87±0.99c	38.89±2.51c	<0.001
arsc	Metal Homeostasis	Arsenic	444.56±25.49a	107.54±11.03b	183.61±7.33c	231.69±6.64d	<0.001
aoxb	Metal Homeostasis	Arsenic	138.08±6.16a	39.96±8.94b	71.64±0.78c	82.02±2.31c	<0.001
mer	Metal Homeostasis	Mercury	373.18±15.44a	102.25±13.98b	169.77±6.14c	204.74±9.23c	<0.001
terc	Metal Homeostasis	Tellurium	427.37±25.24a	108.92±19.02b	206.76±10.45c	223.48±11.67c	<0.001

Table S2 (Continue)

Gene	Gene category	Subcategory	*Galaxea astreata* Mean±SE.	*Porites andrewsi* Mean±SE	*Pavona decussata* Mean±SE	*Porites lutea* Mean±SE	Whole communities P-value[1]
tehb	Metal Homeostasis	Tellurium	102.77±6.7a	28.67±4.93b	40.7±3.02bc	49.24±0.92c	<0.001
urec	Nitrogen	Ammonification	339.05±17.46a	94.13±11.75b	166.17±4.78c	197.72±7.25c	<0.001
gdh	Nitrogen	Ammonification	130.35±5.84a	40.54±4.32b	62.31±1.12c	76.28±3.44d	<0.001
nasa	Nitrogen	Assimilatory N reduction	78.32±3.11a	17.52±3.75b	30.1±3.28c	41.19±0.89d	<0.001
nirb	Nitrogen	Assimilatory N reduction	63.94±5.02a	21.21±2.95b	31.77±2.01c	38.09±1.13c	<0.001
narg	Nitrogen	Denitrification	605±26.58a	152.18±26.55b	285.65±8.25c	351.39±12.97c	<0.001
nosz	Nitrogen	Denitrification	352.54±19.54a	71.49±18.85b	144.86±6.48c	178.33±8.48c	<0.001
nirs	Nitrogen	Denitrification	275.98±15.46a	58.61±7.36b	100.82±4.75c	123.37±5.85c	<0.001
nirk	Nitrogen	Denitrification	248.8±15.25a	58.22±10.14b	84.93±5.97b	125.01±7.15c	<0.001
norb	Nitrogen	Denitrification	69.7±2.45a	15.84±2.6b	27.24±1.47c	38.89±1.06d	<0.001
napa	Nitrogen	Dissimilatory N reduction	92.74±6.22a	14.73±3.37b	31.08±2.6c	41.81±2.79c	<0.001
nitrate reductase	nitrogen	N Assimilation	64.92±4.41a	21.56±3.52b	30.27±0.53bc	37.94±2.52c	<0.001
nifh	Nitrogen	Nitrogen fixation	483.7±37.95a	127.66±18.52b	196.98±7.5bc	230.12±13.19c	<0.001
poba	Organic Remediation	Aromatics	198.37±9.79a	54.41±6.04b	94.91±3.39c	122.9±4.1d	<0.001
catechol	Organic Remediation	Aromatics	148.74±4.23a	40.2±4.78b	77.03±2.84c	95.61±3.98d	<0.001
mdlc	Organic Remediation	Aromatics	142.62±4.94a	42.96±4.27b	74.07±2.74c	91.18±0.3d	<0.001
pcag	Organic Remediation	Aromatics	148.78±7.11a	44.07±5.23b	67.47±2.54c	88.01±0.74d	<0.001
badh	Organic Remediation	Aromatics	139.38±7.45a	35.85±6.41b	63.14±1.92c	80.82±2.14d	<0.001
catechol b	Organic Remediation	Aromatics	144.7±4.54a	27.36±2.97b	56.74±4.57c	78.99±3.15d	<0.001
xylj	Organic Remediation	Aromatics	115.72±3.43a	37.52±4.34b	67.16±1.38c	79.15±2.38d	<0.001
arylest	Organic Remediation	Aromatics	124.28±6.77a	28.44±5.44b	53.87±2.93c	62.1±2.75c	<0.001

Table S2 (Continue)

**Gene**	**Gene category**	**Subcategory**	***Galaxea astreata*** **Mean±SE.**	***Porites andrewsi*** **Mean±SE**	***Pavona decussata*** **Mean±SE**	***Porites lutea*** **Mean±SE**	**Whole communities P-value[1]**
cmci	Organic Remediation	Aromatics	117.5±7.37a	25.61±4.66b	50.74±1.47c	63.59±2.73c	<0.001
xylg	Organic Remediation	Aromatics	97.99±4.39a	34.34±3.2b	53.23±2.25c	68.11±1.85d	<0.001
tfda	Organic Remediation	Aromatics	111.09±5.54a	23.8±5.33b	42.05±1.61c	55.69±2.18d	<0.001
nhh	Organic Remediation	Aromatics	94.25±5.41a	27.48±2.43b	45.54±1.08c	56.45±1.2d	<0.001
bphd	Organic Remediation	Aromatics	96.25±4.49a	26.67±2.29b	40.86±0.77c	47.96±2.27c	<0.001
nitrilase	Organic Remediation	Aromatics	95.32±6.53a	28.6±3.65b	42.67±3.07bc	44.56±3.14c	<0.001
bphc	Organic Remediation	Aromatics	83.42±5.23a	21.56±3.68b	35.76±1.43c	49.25±2.79d	<0.001
catb	Organic Remediation	Aromatics	85.14±3.12a	17.71±2.58b	33.39±0.67c	45.16±1.85d	<0.001
nagg	Organic Remediation	Aromatics	69.06±4.59a	16.76±3.61b	27.66±1.79c	33.87±0.73c	<0.001
one ring 23diox	Organic Remediation	Aromatics	143.64±7.53a	41.02±5.58b	69.44±2.14c	80.93±1.82c	<0.001
bphF1	Organic Remediation	Aromatics	152.73±10.02a	30.81±6.37b	60.45±2.22c	75.39±2.43c	<0.001
mult ring 12DiOx	Organic Remediation	Aromatics	78.52±3.33a	20.28±2.77b	35.72±2.1c	43.06±1.74c	<0.001
one ring 12diox	Organic Remediation	Aromatics	68.45±3.72a	18.7±3b	35.65±1.28c	42.57±1.38c	<0.001
exaa(moxf)	Organic Remediation	Chlorinated solvents	112.7±6.53a	30.98±4.73b	53.98±3.76c	69.21±1.47d	<0.001
dehh109	Organic Remediation	Chlorinated solvents	118.55±5.91a	25.79±5.4b	44.14±3.25c	63.56±2.8d	<0.001
phn	Organic Remediation	Herbicides related compound	180.93±6.79a	57.82±5.01b	85.64±1.38c	107.45±4.75d	<0.001
pcpe	Organic Remediation	Herbicides related compound	122.03±7.1a	34.9±3.51b	51.67±0.97c	68.48±2.81d	<0.001
atzb	Organic Remediation	Herbicides related compound	72.03±4.94a	21.19±2.14b	31.88±1.99c	45.98±0.16d	<0.001
atza	Organic Remediation	Herbicides related compound	71.04±4.8a	17.23±4.35b	33.48±2.03c	34.87±1.56c	<0.001
alkb	Organic Remediation	Other Hydrocarbons	232.01±14.15a	63.22±5.27b	108.27±3.44c	125.79±1.77c	<0.001
chnb	Organic Remediation	Other Hydrocarbons	109.91±6a	38.32±4.34b	53.84±1.74c	64.28±1.76c	<0.001

Table S2 (Continue)

**Gene**	**Gene category**	**Subcategory**	***Galaxea astreata* Mean±SE.**	***Porites andrewsi* Mean±SE**	***Pavona decussata* Mean±SE**	***Porites lutea* Mean±SE**	**Whole communities P-value[1]**
alkk	Organic Remediation	Others	84.45±5.99a	18.75±2.33b	34.87±0.45c	40.67±0.98c	<0.001
linb	Organic Remediation	Pesticides related compound	175.48±8.55a	46.32±4.76b	85.71±4.39c	104.29±2.47d	<0.001
phytase	Phosphorus	Phytic acid hydrolysis	120.92±6.18a	32.82±4.58b	51.39±3.24c	65.56±1.92d	<0.001
ppx	Phosphorus	Polyphosphate degradation	675.1±40.87a	200.9±31.01b	323.6±9.77c	378.95±8.06c	<0.001
ppk	Phosphorus	Polyphosphate synthesis	235.38±13.76a	69.93±7.89b	112.21±3.79c	133.14±3.82c	<0.001
aps apra	Sulfur	adenylylsulfate reductase	113.66±3.97a	32.28±4.24b	56.49±1.74c	62.98±2.94c	<0.001
cysI	Sulfur	Other	155.34±7.45a	42.84±7.1b	72.1±1.49c	90.22±3.24d	<0.001
cysJ	Sulfur	Reduction	230.86±14.14a	72.72±10.71b	119.76±4.31c	141.59±5.92c	<0.001
fccab	Sulfur	Sulfide Oxidation	74.47±4.13a	18.3±1.71b	28.13±3.7bc	31.71±2.01c	<0.001
sqr	Sulfur	Sulfide Oxidation	61±2.13a	20.67±2.55b	29.91±2.06c	33.76±0.82c	<0.001
dsra	Sulfur	sulfite reduction	482.1±36.37a	117.42±20.9b	197.83±13.83c	221.39±9.26c	<0.001
dsrB	Sulfur	sulfite reduction	355.49±22.18a	86.76±12.5b	149.13±6.2c	175.84±4.59c	<0.001
Sir	Sulfur	sulfite reduction	108.14±5.02a	26.24±4.46b	48.83±2.69c	54.28±3.11c	<0.001
soxY	Sulfur	Sulfur Oxidation	144.23±5a	39.14±3.25b	68.69±1.99c	81.47±2.86d	<0.001
B lactamase A	Virulence	Antibiotic resistance	109.16±5.58a	34.9±5.46b	59.07±3.92c	58.74±1.53c	<0.001
b lactamase	Virulence	Antibiotic resistance	79.54±3.62a	20.01±2b	32.51±2.04c	36.75±0.9c	<0.001
ben bcla	virulence	NA	101.57±5.11a	25.26±5.39b	45.1±1.92c	56.61±1.62c	<0.001

[1]P values from the ANOVA among all coral species. [2]Different letters indicate statistical differences at a *P* value of <0.05 among coral species by least-significant-difference (LSD) tests.

Table S3 Significance tests of the differences of the microbial communities between any two coral species in subcategory using Bray–Curtis distances matrices.

	**GA vs PA**	**GA vs PD**	**GA vs PL**	**PA vs PD**	**PA vs PL**	**PD vs PL**
	**F (P)**[a]	**F (P)**	**F (P)**	**F (P)**	**F (P)**	**F (P)**
Carbon degradation	*35.77(0.013)*	*24.16(0.023)*	*17.73(0.014)*	*8.16(0.001)*	*14.24(0.001)*	5.50(0.086)
Carbon fixation	*39.20(0.038)*	*28.99(0.042)*	*17.55(0.003)*	*10.20(0.025)*	*16.22(0.018)*	*6.42(0.004)*
Methane metabolism	*30.04(0.034)*	*47.27(0.019)*	*18.51(0.013)*	8.90(0.072)	*9.58(0.001)*	*5.86(0.001)*
Anammox	*9.99(0.021)*	*7.08(0.001)*	6.84(0.065)	*5.10 (0.042)*	2.35(0.054)	2.83(0.091)
Ammonification	*45.41(0.028)*	*30.87(0.023)*	*16.33(0.026)*	*10.83(0.001)*	*19.12(0.013)*	6.79(0.07)
Nitrogen assimilation	*24.73(0.008)*	35.45(0.052)	*14.72(0.032)*	*4.72(0.009)*	*8.13(0.015)*	*6.69(0.001)*
Assimilatory N reduction	28.48(0.055)	*33.91(0.013)*	*24.82(0.008)*	*7.66(0.037)*	*15.67(0.014)*	*8.69(0.033)*
Denitrification	*30.66(0.001)*	*37.08(0.01)*	*17.21(0.001)*	8.18(0.07)	*13.16(0.011)*	*6.87(0.049)*
Dissimilatory N reduction	18.84(0.067)	*31.17(0.029)*	*18.07(0.001)*	*5.97(0.04)*	*8.75(0.018)*	*7.95(0.001)*
Nitrification	*40.32(0.024)*	*34.68(0.017)*	*13.39(0.03)*	*9.08(0.021)*	*12.91(0.001)*	*7.13(0.001)*
Nitrogen fixation	*32.94(0.001)*	*26.42(0.018)*	*16.46(0.017)*	*6.82(0.001)*	*13.45(0.001)*	*5.99(0.027)*
Adenylysulfate reductase	*39.80(0.048)*	*31.78(0.046)*	*13.94(0.038)*	*9.99(0.013)*	14.15(0.052)	7.03(0.075)
DMSP degradation	*29.70(0.028)*	*23.00(0.036)*	*17.75(0.02)*	*10.32(0.033)*	*16.11(0.044)*	13.32(0.074)
Sulfide oxidation	*50.92(0.043)*	26.38(0.059)	23.28(0.023)	7.24(0.041)	15.33(0.008)	*5.36(0.04)*
Sulfide reduction	*33.73(0.03)*	*25.69(0.016)*	*16.23(0.034)*	*8.36(0.001)*	*12.67(0.001)*	5.05(0.076)
Sulfur oxidation	*52.75(0.026)*	*29.44(0.024)*	*16.32(0.005)*	*12.33(0.001)*	*20.08(0.001)*	*5.21(0.013)*
Phytic acid hydrolysis	*34.87(0.004)*	*22.54(0.035)*	*20.15(0.012)*	*7.37(0.001)*	*15.98(0.01)*	4.70(0.08)
Polyphosphate degradation	*32.45 (0.011)*	*26.48 (0.022)*	*19.34 (0.001)*	*8.98 (0.001)*	*15.09 (0.012)*	*6.68 (0.001)*
Polyphosphate synthesis	*47.61 (0.029)*	*23.63 (0.027)*	*13.37 (0.04)*	*8.51 (0.039)*	14.70 (0.081)	4.61 (0.075)
Aromatics	*41.62 (0.008)*	*29.45 (0.001)*	*18.40 (0.011)*	*9.91 (0.001)*	*17.21 (0.001)*	6.19 (0.09)
Chlorinated solvents	*30.72 (0.015)*	*26.17 (0.035)*	*14.43 (0.01)*	*6.16 (0.021)*	*12.85 (0.001)*	*6.17 (0.001)*
Herbicides related compound	*42.36 (0.034)*	*29.48 (0.001)*	*15.38 (0.005)*	*8.34 (0.001)*	*16.55 (0.001)*	*5.52 (0.001)*
Pesticides related compound	*47.10 (0.021)*	*30.00 (0.021)*	*21.00 (0.001)*	*9.85 (0.001)*	*19.64 (0.005)*	*7.35 (0.019)*
Other hydrocarbons	*48.23 (0.013)*	*32.89 (0.008)*	*19.06 (0.043)*	*10.91 (0.046)*	*15.74 (0.005)*	*6.24 (0.046)*
Arsenic	40.50 (0.068)	26.69 (0.058)	*17.00 (0.036)*	*8.90 (0.02)*	*15.45 (0.024)*	5.90 (0.063)
Mercury	*40.08 (0.041)*	*24.83 (0.001)*	*17.08 (0.001)*	*9.35 (0.014)*	*16.60 (0.001)*	5.92 (0.063)
Tellurium	*31.25 (0.047)*	*23.22 (0.001)*	*16.57 (0.026)*	*8.21 (0.008)*	*12.89 (0.025)*	*5.18 (0.027)*
Chromium	*44.93 (0.034)*	25.97 (0.06)	*14.48 (0.021)*	*10.69 (0.033)*	*15.20 (0.047)*	*2.83 (0.001)*
Copper	48.28 (0.054)	*11.93 (0.028)*	*12.48 (0.038)*	*12.85 (0.016)*	*31.95 (0.001)*	4.56 (0.082)
Silicon	*42.07 (0.009)*	*45.40 (0.022)*	*7.49 (0.001)*	*1.89 (0.034)*	*4.48 (0.001)*	*3.83 (0.001)*

[a]Significance tests were performed by F test based on sequential sums of squares from permutations of the GeoChip hybridization data. P values are of corresponding significance tests. [b]Significant differences ($P<0.05$) are indicated in italic. The full name of each coral species is given in Fig. S1.

Table S4 Biogeochemical cycling categories examined and thenumber of genes sequences detected across all coral samples for each category.

Functional category	No. sequence	Functional category	No. sequence
**Nitrogen cycling**	3272	**Sulfur cycling**	2137
Ammonification[ABCD]	492	Adenylylsulfate reductase[AB]	179
Anammox[B*]	13	DMSP degradation[AB]	51
Nitrogen assimilation[CD]	81	Sulfide oxidation[AB]	145
Assimilatory N reduction[AB]	244	Sulfite reduction[ABC]	1315
Denitrification[ABC]	1661	Sulfur oxidation[B]	278
Dissimilatory N reduction[AB]	174	Others sulfur[B]	169
Nitrification[AB]	51	**Phosphorus cycling**	1107
Nitrogen fixation[AB]	556	Phytic acid hydrolysis[BC]	128
**Carbon cycling**	10915	Polyphosphate degradation[ABC]	724
Carbon degradation	7895	Polyphosphate synthesis[AB]	255
Starch[ABC]	2795	**Organic remediation**	5526
Hemicellulose[ABC]	1041	Aromatics[ABC]	3544
Chitin[ABC]	1028	Herbicides related compound[ABC]	586
Pectin[ABC]	780	Chlorinated solvents[ABC]	376
Cellulose[ABC]	588	Pesticides related compound[ABC]	243
Lignin[ABC]	378	Other hydrocarbons[ABC]	484
Cutin[BC]	212	Other organic remediation[ABC]	293
Vanillin/Lignin[BC]	184	**Metal resistance**	1853
Phospholipids[C]	142	Arsenic[ABC]	738
Terpenes[B]	124	Tellurium[ABC]	580
Carbon fixation	2829	Mercury[ABC]	467
3-hydroxypropionate bicycle[B]	21	Chromium[B]	26
3-hydroxypropionate/4-hydroxybutyrate cycle[AB]	10	Copper[B]	28
Bacterial microcompartments[B]	445	Silicon[D]	14
Calvin cycle[ABD]	1754	**Antibiotic resistance** [ABC]	421
Dicarboxylate/4-hydroxybutyrate cycle[A]	88	**Secondary metabolism**[ABD]	41
Reductive acetyl–CoA pathway[AB]	412		
Reductive tricarboxylic acid cycle[B]	100		
Methane	191		
Methane oxidation[B]	81		
Methanogenesis[AB]	110		

The superscript letters ‘A’ (archaea), ‘B’ (bacteria), ‘C’ (fungi) and 'D' (other eukaryota) indicate the microbial communities identified for each category. *Red marks were first detected in coral holobiont.

Table S5 Environmental parameters of seawater around the coral samples.

	GA	PA	PD	PL	Taxa	Function
	Mean±SE.	Mean±SE	Mean±SE	Mean±SE.	*P*[a]	*P*
Ammonium (μg L^{-1})	39.44±2.33	13.42±0.96	12.95±0.37	34.40±2.27	0.07	***<0.01***
Nitrate (μg L^{-1})	10.70±1.38	46.72±2.82	55.70±0.40	25.16±7.29	0.24	***<0.01***
Nitrite (μg L^{-1})	0.24±0.02	0.51±0.16	2.25±0.18	4.33±0.58	0.07	***<0.01***
Phosphate (μg L^{-1})	16.57±1.99	3.19±0.00	5.30±1.97	7.79±2.79	0.35	***<0.01***
Chlorophyll a (μg L^{-1})	0.77±0.13	1.11±0.26	1.43±0.35	2.86±0.07	***0.04***	***0.01***
PH	8.13±0.01	8.12±0.01	8.11±0.01	8.12±0.02	0.47	0.22
COD (mg L^{-1})	0.19±0.03	0.78±0.16	1.54±0.26	0.31 ±0.02	0.57	0.05
DO (mg L^{-1})	6.01±0.14	6.82±0.04	6.96±0.16	6.06±0.44	***0.03***	***0.03***

[a] Monte Carlo permutation test of environmental attributes with high-throughput sequencing data (Taxa) and GeoChip data (Function). The full name of each coral species is given in Figure S1.

Table S6 Microbial taxa were categorized at the phylum level except for the Proteobacteria.

Functional category	Microbial taxa
Nitrogen cycling	Archaea: Crenarchaeota, Euryarchaeota;
	Bacteria: Acidobacteria, Actinobacteria, Aquificae, Bacteroidetes, Chlamydiae, Chlorobi, Chloroflexi, Cyanobacteria, Deinococcus-Thermus, Fibrobacteres, Firmicutes, Fusobacteria, Gemmatimonadetes, Lentisphaerae, Nitrospirae, Planctomycetes, Alpha-, Beta-, Delta-, Epsilon-, Gamma- Proteobacteria, Spirochaetes, Thermodesulfobacteria, Verrucomicrobia;
	Fungi: Ascomycota
	Other Eukaryota
Carbon cycling	Archaea: Crenarchaeota, Euryarchaeota, Korarchaeota;
	Bacteria: Acidobacteria, Actinobacteria, Aquificae, Bacteroidetes, Chlamydiae, Chlorobi, Chloroflexi, Chrysiogenetes, Cyanobacteria, Deferribacteres, Deinococcus-Thermus, Dictyoglomi, Fibrobacteres, Firmicutes, Fusobacteria, Gemmatimonadetes, Lentisphaerae, Nitrospirae, Planctomycetes, Alpha-, Beta-, Delta-, Epsilon-, Gamma-, Zeta- Proteobacteria, Spirochaetes, Synergistetes, Tenericutes, Thermodesulfobacteria, Thermotogae, Verrucomicrobia;
	Fungi: Ascomycota, Basidiomycota, Neocallimastigomycota
	Other Eukaryota
Sulfur cycling	Archaea: Crenarchaeota calss of Thermoprotei; Euryarchaeota classes of Archaeoglobi, Halobacteria, Methanococci, Methanomicrobia, Methanopyri, and Thermoplasmata
	Bacteria: Acidobacteria, Actinobacteria, Aquificae, Bacteroidetes, Chlorobi, Chloroflexi, Cyanobacteria, Deferribacteres, Deinococcus-Thermus, Fibrobacteres, Firmicutes, Fusobacteria, Nitrospirae, Planctomycetes, Alpha-, Beta-, Delta-, Epsilon-, Gamma- Proteobacteria, Spirochaetes, Synergistetes, Thermodesulfobacteria, Verrucomicrobia;
	Fungi: Ascomycota, Basidiomycota
	Other Eukaryota
Phosphorus cycling	Archaea: Crenarchaeota, Euryarchaeota;
	Bacteria: Acidobacteria, Actinobacteria, Aquificae, Bacteroidetes, Chlamydiae, Chlorobi, Chloroflexi, Cyanobacteria, Deinococcus-Thermus, Firmicutes, Gemmatimonadetes, Nitrospirae, Planctomycetes, Alpha-, Beta-, Delta-, Epsilon-, Gamma- Proteobacteria, Spirochaetes, Synergistetes, Verrucomicrobia;
	Fungi: Ascomycota, Basidiomycota.
Metal homeostasis	Archaea: Crenarchaeota, Euryarchaeota, Korarchaeota;
	Bacteria: Acidobacteriia, Actinobacteria, Aquificae, Bacteroidetes, Chlamydiae, Chlorobi, Chloroflexi, Chrysiogenetes, Cyanobacteria, Deferribacteres, Deinococcus-Thermus, Elusimicrobia, Firmicutes, Gemmatimonadetes, Ignavibacteriae, Nitrospirae, Planctomycetes, Alpha-, Beta-, Delta-, Epsilon-, Gamma-, Zeta- Proteobacteria, Spirochaetes, Synergistetes, Verrucomicrobia
	Fungi: Ascomycota
	Other Eukaryota
Organic Remediation	Archaea: Crenarchaeota, Euryarchaeota:
	Bacteria: Acidobacteriia, Actinobacteria, Aquificae, Bacteroidetes, Chlorobi, Chloroflexi, Cyanobacteria, Deinococcus-Thermus, Fibrobacteres, Firmicutes, Nitrospirae, Planctomycetes, Alpha-, Beta-, Delta-, Epsilon-, Gamma- Proteobacteria, Spirochaetes, Thermodesulfobacteria, Verrucomicrobia;
	Fungi: Ascomycota, Basidiomycota
	Other Eukaryota

References

1 Zhou, J. *et al.* Reproducibility and quantitation of amplicon sequencing-based detection. *ISME J.* **5**, 1303-1313 (2011).

2 Caporaso, J. G. *et al.* Global patterns of 16S rRNA diversity at a depth of millions of sequences per sample. *Proc. Natl. Acad. Sci. U. S. A.* **108**, 4516-4522 (2011).

3 Caporaso, J. G. *et al.* Ultra-high-throughput microbial community analysis on the Illumina HiSeq and MiSeq platforms. *ISME J.* **6**, 1621-1624 (2012).
